# Dermal Filler‐Induced Alopecia: A Case Report and Literature Review

**DOI:** 10.1111/jocd.16684

**Published:** 2024-11-20

**Authors:** Salma Albargawi, Khalid Nabil Nagshabandi, Asem Shadid

**Affiliations:** ^1^ Department of Dermatology, College of Medicine Imam Mohammad Bin Saud Islamic University Riyadh Saudi Arabia; ^2^ Department of Dermatology, College of Medicine King Saud University Riyadh Saudi Arabia; ^3^ Department of Dermatology King Fahad Medical City Riyadh Saudi Arabia

**Keywords:** aesthetic medicine, alopecia, autologous fat, calcium hydroxyapatite, cosmetic, filler complication, hair loss, hyaluronic acid, hyaluronidase, vascular occlusion

## Abstract

**Background:**

Dermal filler‐induced alopecia is a rare yet significant complication of aesthetic procedures primarily associated with vascular occlusion and subsequent tissue ischemia. Hyaluronic acid (HA) fillers, though widely used for facial rejuvenation, can lead to adverse outcomes such as skin necrosis and hair loss, particularly in high‐risk areas like the temples and glabella.

**Objective:**

This case report aims to highlight the clinical presentation, diagnostic approach, and multidisciplinary management of filler‐induced alopecia, contributing to the existing literature with a comprehensive review of previously reported cases.

**Method:**

A 21‐year‐old female presented with localized skin necrosis and alopecia four days after receiving 7 mL of HA filler injections in the temples, tear trough, and eyebrow glabella regions. Trichoscopy revealed follicular dropout and white dots, consistent with ischemic hair loss. Treatment included hyaluronidase injections (1500 units), intralesional corticosteroids, topical minoxidil, and CO_2_ laser therapy. Over 1 year of follow‐up, the patient achieved complete hair regrowth and resolution of facial scarring.

**Results:**

Only 16 cases of filler‐induced alopecia have been documented, predominantly involving HA fillers. This case underscores the importance of early recognition and intervention with hyaluronidase to mitigate ischemic damage. The multidisciplinary management approach employed here demonstrates the potential for full cosmetic recovery.

**Conclusion:**

Filler‐induced alopecia, though rare, necessitates heightened awareness among dermatologists and aesthetic practitioners. Adhering to recommended injection techniques and dosages, alongside the judicious use of ultrasound guidance, can minimize risks and improve patient safety.

## 1 Introduction

Cosmetic facial injections, including hyaluronic acid (HA) fillers, is currently one of the most prevalent and widely employed nonsurgical cosmetic procedures aimed at enhancing facial volume and contour [1]. However, the occurrence of vascular occlusion as a consequence of HA filling is not infrequent and is correlated with a spectrum of severe complications and adverse events, including skin necrosis, permanent blindness, and ischemic stroke [1]. Given the complexity and variability of facial anatomy, injecting fillers into less commonly treated regions may introduce unexpected risks. Localized alopecia subsequent to HA filling is a recently documented complication, with vascular compromise having been identified as its primary pathological mechanism [2]. In addition to HA fillers, complications have been reported with other fillers such as calcium hydroxyapatite (CaHA) and autologous fat injections. The pathophysiology appears to be similar, involving vascular compromise and subsequent tissue ischemia to the skin and hair follicles. The clinical utility of hyaluronidase has been established as a highly effective approach in addressing complications arising from perfusion blockade within or around HA injection sites. Given the diverse levels of resistance to ischemic injury exhibited by various organs and tissues, the temporal parameters for the optimal administration of hyaluronidase display variability [3]. The use of ultrasound guidance during filler injections has emerged as an effective technique in reducing the risk of vascular compromise, particularly in high‐risk areas like the temples and periorbital region [4, 5].

Filler‐induced alopecia and skin necrosis are rare and devastating complications that require prompt diagnosis and intervention. To the best of our knowledge, only (16) cases, including the one reported herein, of post filler‐induced hair loss have been reported in the literature [6, 7, 8, 9, 10, 11, 12, 13, 14, 15]. Herein, we report a rare instance of a 21‐year‐old female who developed HA injection‐induced alopecia and skin necrosis 4 days post filler injection of temples, tear troughs, and eyebrow glabella.

## 2 Case Presentation

**FIGURE 1:**
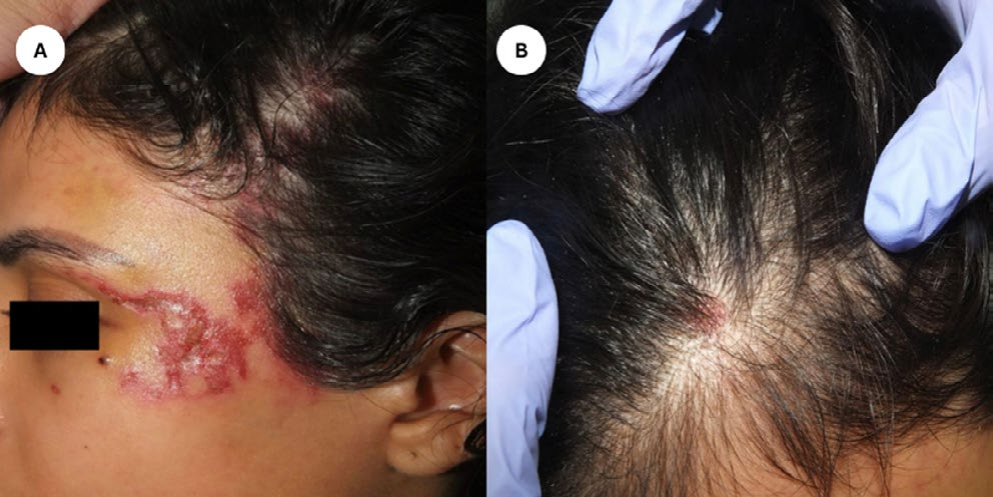
(A) Distinct, red, eroded, and partially ulcerated patch in a reticulated pattern observed on the left temporal area. (B) Additional few tender, well‐defined, red patches on the scalp.

**FIGURE 2:**
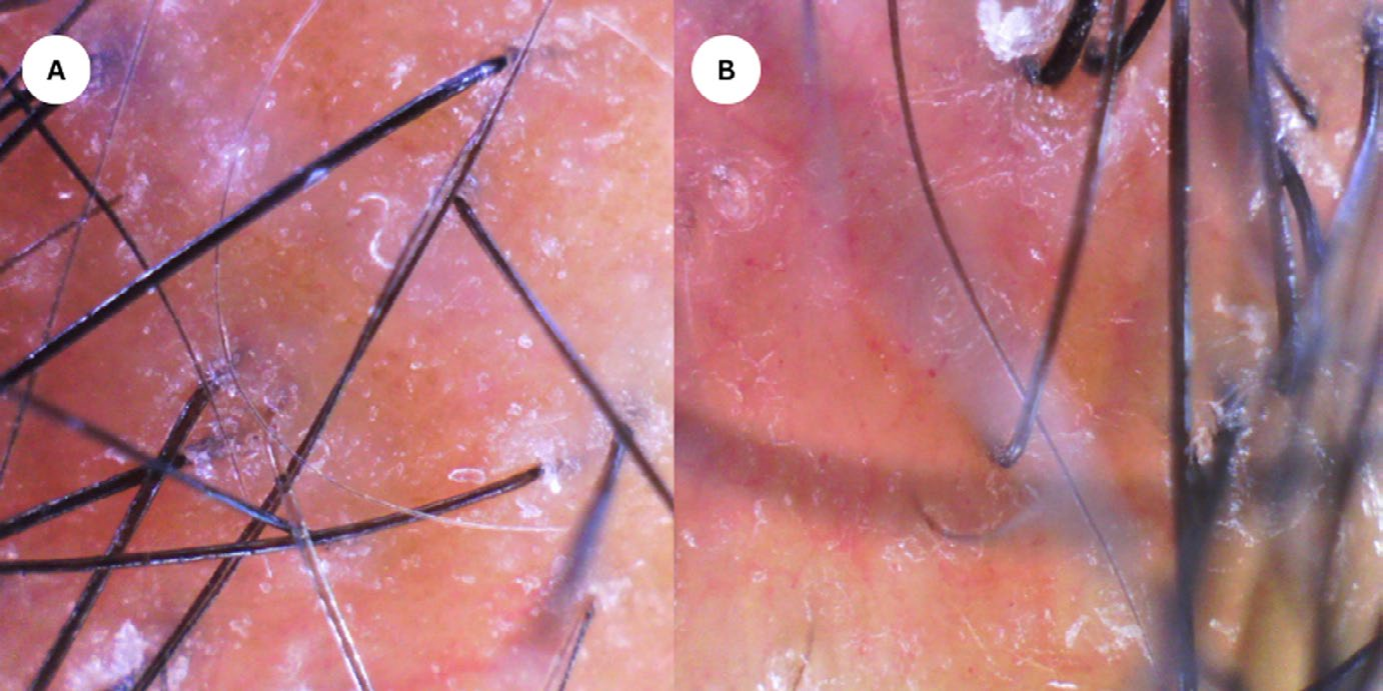
(A, B) Scalp trichoscopy showed follicular dropout and a notable extent of hair loss from the follicles, accompanied by the presence of white dots.

**FIGURE 3:**
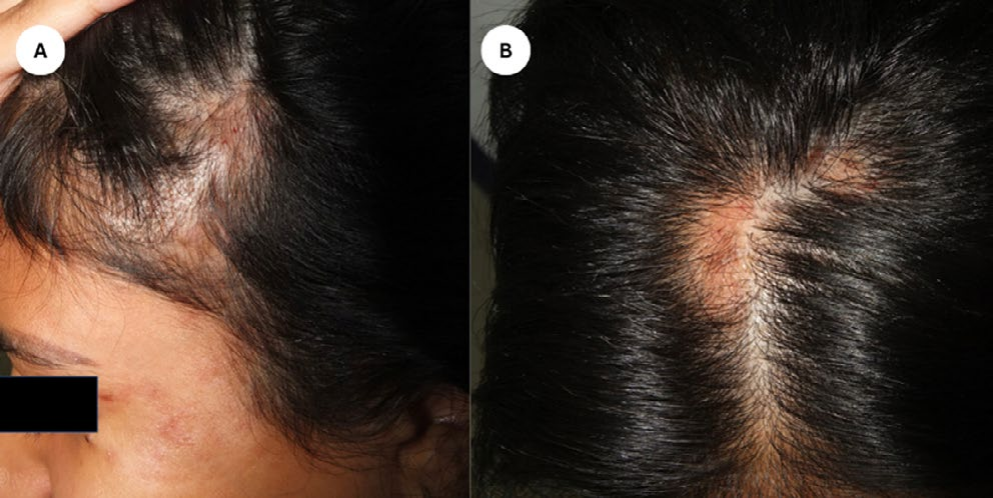
(A, B) Approximately a month after filler injection, examination of the scalp revealed patches of hair loss in regions corresponding to the injection sites.

**FIGURE 4:**
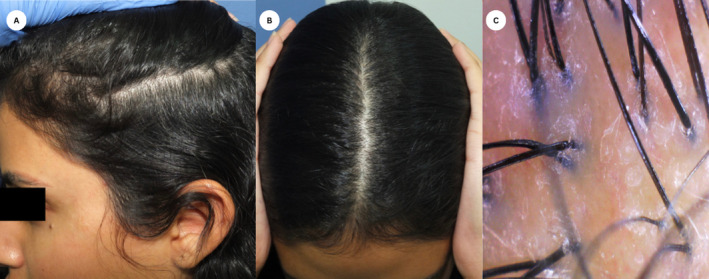
One‐year follow‐up. (A) No residual scarring left on the face. (B) Complete hair regrowth evident clinically. (C) Scalp trichoscopy revealed follicle regrowth.

A 21‐year‐old female presented with painful lesions on the left temporal, tear trough, forehead, and eyebrows 4 days postinjection with 7 mL of HA for facial enhancement. The lesions rapidly progressed, accompanied by localized hair loss and skin necrosis. Clinical examination revealed a well‐defined erythematous eroded and partially ulcerated plaque in a reticulated pattern over left temporal and a few tender well‐defined erythematous plaques over the scalp (Figure 1). The patient had no significant medical or dermatological history, and there were no prior episodes of hair loss before receiving the HA filler injections. A clinical impression of skin necrosis due to intravascular injection was appreciated and a skin necrosis management protocol was implemented promptly using hyaluronidase injection (1500 units). Trichoscopic assessment of the scalp was performed using polarized light and revealed follicular dropout and a significant amount of follicular hair loss with white dots (Figure 2). Although histopathologic confirmation of filler‐induced alopecia would have provided additional diagnostic certainty, the patient declined further invasive procedures. A diagnosis of filler‐induced alopecia was established based on the clinical presentation and the temporal relationship with the HA injection. We commenced intralesional triamcinolone acetonide (5 mg/mL) injections into the affected scalp areas to mitigate the inflammatory response and promote hair regrowth. The patient received three intralesional corticosteroid injections at 4‐week intervals. Moreover, the patient was instructed to apply topical 2% minoxidil twice daily to enhance hair follicle stimulation. The patient also underwent CO_2_ laser therapy sessions to promote skin healing and reduce inflammation on the affected facial areas. She underwent four sessions of CO_2_ laser therapy, spaced 2 weeks apart, targeting the areas of skin necrosis. Nearly 1‐month post filler injection, the facial lesions showed improvement with laser therapy, but scalp examination showed alopecia patches in areas corresponding to the injected sites (Figure 3). The patient responded well to the combined treatment approach. Lesions showed gradual improvement in terms of pain, erythema, and necrosis. Hair regrowth on the scalp was observed over several months. Nearly 1 year of follow‐up assessments, the patient reported a satisfactory cosmetic outcome with no residual scarring left on the face, and complete hair regrowth evident by clinical and trichoscopy examination (Figure 4). The patient was regularly monitored through biweekly follow‐up visits. Compliance with topical minoxidil and was evaluated during each visit, and the patient reported consistent adherence to the prescribed regimen.

## 3 Discussion

The aging process manifests in various external changes, prompting individuals to pursue skin rejuvenation as a means to attenuate these effects. Volume depletion is an initial indicator of aging, contributing to a facial aesthetic characterized by a skeletal and haggard appearance. HA filling serves as a dependable method for restoring volume in the temple region, enhancing the overall youthful contours of the face [1]. However, the occurrence of vascular complications related to HA filling, while infrequent in morbidity, may result in unfavorable consequences, including skin tissue necrosis. In clinical settings, skin necrosis typically arises from inadvertent intravascular injection or external compression and pressure due to localized excessive HA filling [2]. Given HA's considerable hygroscopic nature, it possesses the capacity to absorb a substantial volume of water, consequently significantly escalating hydrostatic pressure within the local interstitium [1, 16]. Numerous researchers have advocated for a recommended dosage of HA in temple filling, typically ranging from 0.5 to 3 mL per temple [2]. In our case, the patient underwent an injection of 7 mL of HA in each temple, surpassing the recommended range, thereby elevating the potential for compression‐induced tissue ischemia. Ultrasound, particularly high‐frequency ultrasound (HFUS), has emerged as a crucial tool in minimizing and managing vascular complications associated with dermal filler injections. These complications typically result from unintentional filler injection into or around blood vessels, leading to occlusion or compression of blood flow. Ultrasound offers real‐time imaging that significantly improves the precision and safety of filler injections, thereby reducing the likelihood and severity of these adverse events [4, 5].

The clinical manifestations, histopathological characteristics, and progression of alopecia induced by fillers exhibit notable similarities to those associated with pressure‐induced alopecia [9]. However, individuals experiencing hair loss as a result of filler injections typically exhibit a shorter onset time for alopecia postinjection compared with those affected by pressure‐induced alopecia. Furthermore, the incidence rates of skin necrosis and permanent hair loss are significantly elevated in cases of filler‐induced alopecia [9]. This suggests a potential shared etiology between filler‐induced and pressure‐induced alopecias, specifically involving vascular compromise, leading to local tissue hypoperfusion and hypoxia. However, the varying degrees of ischemic damage indicate distinct manifestations of the condition. In our case, we diagnosed intravascular occlusion as the primary mechanism, given the rapid onset of alopecia postinjection, the presence of necrotic skin changes, and the distribution of alopecia along the injection sites, which is consistent with vascular compromise.

The occurrence of scalp necrosis leading to subsequent alopecia following filler injection represents a complication that, despite having been previously documented, is still considered an unusual sequelae. Only 16 cases, including the one documented herein, have been reported to date. A summary and comparison of the reported cases of filler‐induced alopecia are provided in (Table 1) [6, 7, 8, 9, 10, 11, 12, 13, 14, 15]. The patients included in the table range in age from 21 to 71 years, with a significant concentration of middle‐aged women (more commonly in women in their 30s to 60s). Out of the 16 documented cases only one male patient is represented, highlighting a predominance of female instances. Hyaluronic acid (HA) is the most common filler type used in these cases, being present in 13 out of 16 cases. HA is widely used due to its temporary nature and fewer complications compared with permanent fillers. Two cases involve CaHA, a longer‐lasting filler, with one patient experiencing ongoing alopecia. Autologous fat filler injection (permanent) was only used in one case, suggesting that while it is a rare cause, it may still lead to alopecia. The temples are the most frequently reported area of injection, followed by regions such as the scalp, superciliary arch, and cheekbone. The temporal area is particularly susceptible due to its proximity to blood vessels that may affect hair follicles. In our case, HA filler was injected into multiple regions, including the temples, tear trough, eyebrow, and glabella, which may reflect a broader distribution of filler‐related alopecia risk zones. The amount of filler varied widely, with some cases reporting as much as 7 mL/side, and others using more moderate amounts of 1 to 3 mL per side. Skin necrosis was present in most of the cases (13 out of 16), suggesting a strong link between tissue damage and subsequent alopecia. The onset of alopecia ranged from 1 week to 1 month after filler injection, with most cases manifesting within 2 weeks. Most cases reported the onset of alopecia within 1 to 2 weeks after filler injection. For example, the cases by Yang Q et al. (2017), Khunkhet S et al. (2019), and Asz‐Sigall D et al. (2019) all experienced alopecia within this time frame [5, 7, 8]. This early onset is often associated with complications like necrosis and vascular compromise, where filler material may block or compress blood vessels, leading to localized ischemia and subsequent hair follicle damage. A few cases reported a delayed onset of alopecia, occurring 3 to 4 weeks after the injection. For instance, Park MJ et al. (2020) and Liu RF et al. (2018) noted the onset of alopecia approximately 1 month after the procedure [6, 10]. Delayed onset may be due to slower progressive tissue damage or subclinical inflammation that becomes apparent over time. In our report, the onset of alopecia occurred 1 month after filler injections, similar to some of the delayed onset cases in the literature. The onset of alopecia does not seem to correlate directly with the type of filler used, as both hyaluronic acid and calcium hydroxyapatite fillers resulted in alopecia within a similar time frame. Cases with necrosis tended to show early‐onset alopecia. This rapid hair loss is likely due to the acute compromise of blood flow to hair follicles, leading to ischemia and follicular death within a short period after the filler injection. The majority of cases with necrosis reported alopecia onset within 1 to 2 weeks.

**TABLE 1:** Summary overview and comparison of reported cases of filler‐induced alopecia [6, 7, 8, 9, 10, 11, 12, 13, 14, 15].

Case (year)	Age (years) /gender	Type of filler	Area	Amount	Necrosis	Onset of alopecia	Site of alopecia	Management	Recovery
Gan SD et al. [6] (2013)	58/Female	HA	Temples	6 mL/side	−	2 weeks	Right temple	Intralesional triamcinolone (5 mg/mL) twice	3 months: 50% hair regrowth + scarring alopecia
Yang Q et al. [7] (2017)	27/Female	HA	Temples	6.5 mL/side	+	2 weeks	Left temporo‐parietal	Hyaluronidase (bFGF) gel + 2% minoxidil spray	7 months: Regrowth of hair + scarring alopecia
Liu RF et al. [8] (2018)	44/Female	CaHA	Scalp	NA	−	1 month	Frontal scalp	Not treated	6 months: Complete hair regrowth + no scarring
Khunkhet S et al. [9] (2019)	36/Female	Autologous fat	Temples	2 mL/side	+	2 weeks	Right temporo‐parietal	5% minoxidil lotion	6 months: Restoration of full hair density + no scarring
Asz‐Sigall D et al. [10] (2019)	30/Female	HA	Superciliary arch	NA	+	1 week	Right frontoparietal scalp	Oral amoxicillin and clavulanic acid BID (partial response and improvement of erythema) (initial impression of skin infection) + intralesional triamcinolone (10 mg/mL) +hyaluronidase (50 units) + oral acetylsalicylic acid (100 mg) + minoxidil 2% spray	1 week: after the hyaluronidase injection: alopecic patch stabilized and stopped expanding + swelling, erythema, and pain decreased 2 months: Hair regrowth + no scarring
Park GH et al. [11] (2019)	58/Female	HA	Temples	NA	+	1 week	Right temple	Hyaluronidase + intralesional triamcinolone (5 mg/mL) twice + 5% minoxidil spray	5 months: Complete hair regrowth + no scarring
Park MJ et al. [12] (2020)	59/Female	HA	Scalp	3 mL	−	1 month	Frontal and mid‐scalp	Intralesional triamcinolone (5 mg/mL)	6 months: Complete hair regrowth + no scarring
van den Elzen H et al. [13] (2022)	54/Female	HA	Right lateral cheekbone	0.75 mL	+	11–18 days	Right temporoparietal scalp	Hyaluronidase (60 units)	9 months: Complete hair regrowth + no scarring
Landau M et al. [14] (2023)	61/Female	HA	Posterior temples	1 mL/side	+	10 days	Central scalp	Hyaluronidase (7500 units) + platelet‐rich plasma sessions (biweekly)	3 months: Hair regrowth started
	44/Female	HA	Posterior temples	0.5 mL/side	+	9 days	Left temporoparietal scalp	Hyaluronidase (69 000 units) + HA extraction + platelet‐rich plasma sessions topical and oral minoxidil + topical finasteride	4 months: Hair regrowth started 12 months: Complete hair regrowth, except for a small alopecic patch
	41/Female	HA	Posterior temples	1 mL/side	−	14 days	Central scalp	Painkillers	3 months: Hair regrowth started 6 months: Complete hair regrowth, except for a small alopecic patch
	71/Female	CaHA	Posterior temples	1 mL	+	3 weeks	Right temporoparietal scalp	Oral prednisone 40 mg + topical minoxidil 5%	Ongoing alopecia
	36/Female	HA	Posterior temples	1 mL/side	+	15 days	Left parietal scalp	Hyaluronidase	1.5 months: Hair regrowth started
	52/Male	HA	Posterior temples	1.2 mL/side	+	14 days	Right parietal scalp	Hyaluronidase (300 units) + HA extraction	Alopecia lasted 6 months
Pearce J et al. [15] (2023)	37/Female	HA	Temples	NA	+	NA	Frontotemporoparietal scalp	Hyaluronidase + oral and topical corticosteroids + hydroxychloroquine	NA + scarring alopecia patch
Albargawi S et al. (Our case) (2024)	21/Female	HA	Temples Tear trough Eyebrow glabella	7 mL/side	+	1 month	Left frontal and temporal scalp	Hyaluronidase (1500 units) + intralesional triamcinolone (5 mg/mL) twice + 2% minoxidil spray	1 year: Complete hair regrowth + no scarring

Abbreviations: bFGF, basic fibroblast growth factor; CaHA, calcium hydroxyapatite; HA, hyaluronic acid; mg, milligram; mL, milliliter; NA, not applicable.

The temporal and parietal scalp regions of the scalp were most commonly affected sites of alopecia, followed by the frontal and central scalp. This is consistent with the fact that many filler injections in the reviewed cases were administered in the temples or cheekbone area, regions with a rich network of blood vessels. The temporal area is particularly vulnerable due to the close proximity of the superficial temporal artery and other small vascular branches that supply the hair follicles [17]. Several cases also involved the frontal scalp, especially when fillers were injected near the forehead or superciliary arch [8, 10]. A few cases involved alopecia in the central scalp or midscalp regions, which are less commonly injected directly but can be affected through the vascular compromise from nearby injections [12]. Various treatment options were used, with hyaluronidase being the most common intervention. This enzyme helps dissolve HA fillers and has shown efficacy in reversing alopecia and skin necrosis in many cases. Other management options include intralesional triamcinolone, minoxidil (both topical and oral), platelet‐rich plasma (PRP), and in some cases, surgical extraction of the filler. Oral corticosteroids (prednisolone) were used in more complex cases with severe inflammation or necrosis. A critical distinction in outcomes was the development of scarring alopecia, where hair follicles are permanently damaged, leading to irreversible hair loss in the affected area. Scarring Alopecia was reported in fewer cases but is significant because it indicates permanent hair follicle damage. For instance, Gan SD et al. (2013) and two other instances reported scarring despite partial hair regrowth [4, 5, 13]. But most cases demonstrated nonscarring alopecia, with full hair recovery observed. For most patients, long‐term recovery appeared promising, with no lasting alopecia in the majority of cases. Even in cases where alopecia persisted for several months, such as the instance reported herein, complete regrowth without scarring was achieved at 1 year, showcasing the potential for full recovery with proper management.

A recent review by Kroumpouzos G et al. provides a comprehensive guide for using hyaluronidase in managing dermal filler complications. For noninflammatory nodules, the recommended dosage is between 5 and 15 IU, and adjustments are made based on filler properties and patient response. Vascular occlusions require a higher dose, with protocols recommending 300–1500 IU, depending on the size of the ischemic area and the type of hyaluronic acid filler used. The authors also suggest using bacteriostatic saline for reconstitution and avoiding lidocaine in reconstitution, as it may interfere with hyaluronidase's enzymatic activity. For delayed onset nodules or granulomas, the combination of hyaluronidase and oral antibiotics is suggested, with recommended doses starting from 30 to 300 IU [18]. There is no widely established or standardized guideline specifically for the management of filler‐induced ischemic alopecia. However, the management of this condition typically follows principles used in treating ischemia and vascular occlusions from dermal fillers. The primary management approach for filler‐induced alopecia focuses on early intervention to restore blood flow and prevent permanent follicular and skin damage. Hyaluronidase, particularly utilized for dissolving hyaluronic acid (HA) fillers, is the most commonly used intervention and has demonstrated significant efficacy in most reported cases. Most patients report substantial hair regrowth within a few months following hyaluronidase treatment. Other treatment options include the use of minoxidil, both topical and oral, topical DHT blockers (finasteride), and platelet‐rich plasma (PRP), which may support hair restoration. In more severe cases involving significant inflammation or necrosis, additional management strategies such as intralesional triamcinolone and oral corticosteroids (e.g., prednisolone) are employed. For cases where fillers need to be physically removed, surgical extraction is considered. In our case, the patient also underwent CO_2_ laser therapy to address facial skin necrosis, receiving four sessions spaced 2 weeks apart. The CO_2_ laser sessions aimed to enhance skin healing and reduce inflammation in the affected facial areas. noticeable improvement was observed in the facial lesions 1 month post laser therapy. These combined approaches aim to reverse alopecia and follicular damage and also mitigate the risks of lasting skin scarring.

The intricate interplay between aging, facial volume restoration through dermal filling, and the associated risks of vascular complications underscores the need for a meticulous and evidence‐based approach in aesthetic procedures. Awareness of potential adverse outcomes, adherence to recommended dosages, and ongoing research efforts are crucial in navigating the delicate balance between achieving youthful aesthetics and mitigating unintended consequences in the realm of cosmetic interventions.

## 4 Conclusion

Filler‐induced ischemic alopecia and skin necrosis are rare but serious complications. Continued vigilance and awareness among dermatologists and cosmetic practitioners are crucial for minimizing the risk of permanent alopecia in patients undergoing filler‐based procedures. Early recognition and a multidisciplinary approach, including hyaluronidase, corticosteroid injection, and topical or oral minoxidil, can contribute to successful management including hair regrowth and cosmetic skin recovery.

## Author Contributions

S.A. manuscript writing, history and physical examination sections, critical revisions, approved the final version for submission. K.N.N. manuscript writing, discussion and conclusion sections, intellectual input, approved the final version for publication. A.S. manuscript writing, case presentation and management sections, critical feedback, approved the final version for submission.

## Ethics Statement

The authors have nothing to report.

## Consent

Written informed consent was obtained from the patient for publication of the details of their medical case and any accompanying images.

## Conflicts of Interest

The authors declare no conflicts of interest.

## Data Availability

All data generated or analyzed during this study are included in this article. Further enquiries can be directed to the corresponding author.
